# New Coleoptera records from New Brunswick, Canada: Silvanidae and Laemophloeidae

**DOI:** 10.3897/zookeys.179.2600

**Published:** 2012-04-04

**Authors:** Reginald P. Webster, Jon D. Sweeney, Ian deMerchant

**Affiliations:** 1 Natural Resources Canada, Canadian Forest Service, Atlantic Forestry Centre, 1350 Regent St., P.O. Box 4000, Fredericton, NB, Canada E3B 5P7

**Keywords:** Silvanidae, Laemophloeidae, *Leptophloeus*, new records, Canada, New Brunswick

## Abstract

One species of Silvanidae, *Silvanus muticus* Sharp, is newly recorded from New Brunswick, Canada and the Maritime provinces; *Ahasverus longulus* (Blatchley) is re-instated to the faunal list of the province, and we report the first recent provincial records of *Dendrophagus cygnaei* Mannerheim. Five species of Laemophloeidae (*Charaphloeus convexulus* (LeConte), *Charaphloeus* undescribed species (near *adustus*), *Leptophloeus angustulus* (LeConte), *Placonotus zimmermanni* (LeConte), and an undescribed *Leptophloeus* species) are added to the faunal list of New Brunswick. Collection data, bionomic data, and distribution maps are presented for all these species.

## Introduction

This paper treats new records from New Brunswick of two related families of beetles, the Silvanidae and the Laemophoeidae. The Silvanidae, Cucujidae, and Laemophloeidae of Atlantic Canada were reviewed by Majka (2008). Five species of Silvanidae were reported from New Brunswick by Majka (2008), three as new to the province, and one species was removed from the provincial list. Three species of Laemophloeidae were reported from the province, two as new (Majka 2008). Intensive sampling in New Brunswick by the first author since 2003 and records obtained from by-catch samples in Lindgren funnel traps set in various New Brunswick forest sites from 2008–2011 have yielded additional new provincial records in the above families. The purpose of this paper is to report on these new records. A brief synopsis of each family is included in the results below.

## Methods and conventions

The following records are based, in part, on specimens collected during a general survey by the first author to document the Coleoptera fauna of New Brunswick and from by-catch samples collected in Lindgren funnel traps during a study testing attractants for detection of longhorn beetle species. Additional records were obtained from specimens contained in the collection belonging to Natural Resources Canada, Canadian Forest Service - Atlantic Forestry Centre, Fredericton, New Brunswick.

### Collection methods

Various methods were employed to collect the species reported in this study. Details are outlined in Webster et al. (2009, Appendix). Many specimens were also collected from Lindgren 12-unit funnel trap samples during a study to develop a general attractant for the detection of invasive species of Cerambycidae. These traps mimic tree trunks and are often effective for sampling species of Coleoptera that live in microhabitats associated with standing trees (Lindgren 1983). See Webster et al. (in press) for details of the methods used for deployment of funnel traps and sample collection. A description of the habitat was recorded for all specimens collected during this survey. Locality and habitat data are presented exactly as on labels for each record. This information, as well as additional collecting notes, is summarized and discussed in collection and habitat data section for each species.

### Distribution

Distribution maps, created using ArcMap and ArcGIS, are presented for each species in New Brunswick. Every species is cited with current distribution in Canada and Alaska, using abbreviations for the state, provinces, and territories. New records for New Brunswick are indicated in bold under Distribution in Canada and Alaska. The following abbreviations are used in the text:

Acronyms of collections examined or where specimens reside referred to in this study are as follows:

AFC Atlantic Forestry Centre, Natural Resources Canada, Canadian Forest Service, Fredericton, New Brunswick, Canada

CNC Canadian National Collection of Insects, Arachnids and Nematodes, Agriculture and Agri-Food Canada, Ottawa, Ontario, Canada

NBM New Brunswick Museum, Saint John, New Brunswick, Canada

RWC Reginald P. Webster Collection, Charters Settlement, New Brunswick, Canada

**Table:** 

**AK**	Alaska	**MB**	Manitoba
**YT**	Yukon Territory	**ON**	Ontario
**NT**	Northwest Territories	**QC**	Quebec
**NU**	Nunavut	**NB**	New Brunswick
**BC**	British Columbia	**PE**	Prince Edward Island
**AB**	Alberta	**NS**	Nova Scotia
**SK**	Saskatchewan	**NF & LB**	Newfoundland and Labrador

## Results

### Species accounts

All records below are species newly recorded for New Brunswick, Canada, unless noted otherwise (additional records). Species followed by ** are newly recorded from the Maritime provinces (New Brunswick, Nova Scotia, Prince Edward Island) of Canada.

The classification of the Silvanidae and Laemophloeidae follows Bouchard et al. (2011).

**Table 1.** Species of Silvanidae and Laemophloeidae recorded from New Brunswick, Canada.

Family Silvanidae Kirby
Subfamily Brontinae Blanchard
Tribe Brontini Blanchard
*Dendrophagus cygnaei* Mannerheim
Subfamily Silvaninae Kirby
*Ahasverus longulus* (Blatchley)**
*Nausibius clavicornis* (Kugelann)
*Oryzaephilus mercator* (Fauvel)
*Oryzaephilus surinamensis* (Linnaeus)
*Silvanus bidentatus* (Fabricius)
*Silvanus muticus* Sharp**
Family Laemophloeidae Ganglbauer
*Charaphloeus* convexulus (LeConte)*
*Charaphloeus* undescribed species (near *adustus*)*
*Cryptolestes pusillus* (Schönherr)
*Laemophloeus biguttatu*s (Say)
*Laemophloeus fasciatus* Melsheimer
*Placonotus zimmermanni* (LeConte)*
*Leptophloeus angustulus* (LeConte)*
*Leptophloeus undescribed* species **

**Notes:** *New to province, **New to Maritime provinces.

#### Family Silvanidae Kirby, 1837

Thomas (2002a) presented a general overview of the Family Silvanidae (silvanid flat bark beetles) of North America. Little is known about the biology and immature stages of most species of this family. Brontinae are usually found under bark, where adults and larvae likely feed on ascomycete and other fungi (Crowson and Ellis 1969); the Silvaninae are subcortical or live in leaf litter or soil, and feed on fungi (Thomas 2002a). Some species are stored-product pests. Bousquet (1991) reported three species of Silvanidae, *Silvanus bidentatus* (Fabricius), *Ahasverus longulus* (Blatchley), and *Oryzaephilus mercator* (Fauvel) from New Brunswick. Majka (2008), in a review of the flat bark beetles of Atlantic Canada, added another three species (*Dendrophagus cygnaei* Mannerheim, *Nausibius clavicornis* (Kugelann), *Oryzaephilus surinamensis* (Linnaeus)) and removed *Ahasverus longulus* from the faunal list due to a lack of supporting voucher specimen or other published records. In this account, *Silvanus muticus* Sharp is newly recorded from New Brunswick and the Maritime provinces, *Ahasverus longulus* is re-instated to the faunal list, and we report the first recent records of *Dendrophagus cygnaei* from the province (Table 1).

#### Subfamily Brontinae Blanchard, 1845. Tribe Brontini Blanchard, 1845

##### Dendrophagus cygnaei

Mannerheim, 1846

http://species-id.net/wiki/Dendrophagus_cygnaei

Map 1

###### Material examined.

**Additional New Brunswick records. Carleton Co.**, Richmond, near Hovey Hill P.N.A. (Protected Natural Area), 46.1155°N, 67.7631°W, 10.V.2005, R. P. Webster, clear-cut (hardwood forest), under bark of *Populus* sp. (2, RWC); Jackson Falls, Bell Forest, 46.2200°N, 67.7231°W, 19–27.VI.2008, 5–12.VII.2008, R. P. Webster, mature hardwood forest, Lindgren funnel traps (3, AFC, RWC); same locality and habitat data but 23–28.IV.2009, 14–20.V.2009, R. Webster & M.-A. Giguère, Lindgren funnel traps (5, AFC). **Charlotte Co.**, 10 km NW of New River Beach, 45.2110°N, 66.6170°W, 30.IV-17.V.2010, R. Webster & C. MacKay, old growth eastern white cedar forest, Lindgren funnel trap (1, AFC). **Queens Co.**, Cranberry Lake P.N.A, 46.1125°N, 65.6075°W, 24.IV-5.V.2009, 21–27.V.2009, R. Webster & M.-A. Giguère, old red oak forest, Lindgren funnel traps (6, AFC). **Restigouche Co.**, Dionne Brook P.N.A., 47.9030°N, 68.3503°W, 30.V–15.VI.2011, M. Roy & V. Webster, old-growth northern hardwood forest, Lindgren funnel traps (2, AFC, NBM); same locality and collectors but 47.9064°N, 68.3441°W, 31.V–15.VI.2011, old-growth white spruce and balsam fir forest, Lindgren funnel traps (3, AFC, NBM). **Sunbury Co.**, Acadia Research Forest, 45.9866°N, 66.3841°W, 8–13.V.2009, 13–19.V.2009, 19–25.V.2009, R. Webster & M.-A. Giguère, mature (110-year-old) red spruce forest with scattered red maple and balsam fir, Lindgren funnel traps (4, AFC). **York Co.**, Charters Settlement, 45.8342°N, 66.7452°W, 23.IV.2004, R. P. Webster, mixed forest, under bark of sugar maple (1, RWC); Charters Settlement, 45.8395°N, 66.7391°W, 19.V.2007, R. P. Webster, mixed forest, under bark of large *Populus* sp log (2, RWC); Canterbury, 45.8920°N, 67.6592°W, 8.VI.2004, D. Sabine & R. Webster, hardwood forest, under bark (1, RWC); 15 km W of Tracy off Rt. 645, 45.6848°N, 66.8821°W, 22–25.IV.2009, 25.IV-4.V.2009, 7–14.VII.2009, R. Webster & M.-A. Giguère, old red pine forest, Lindgren funnel traps (8, AFC, RWC); 14 km WSW of Tracy, S of Rt. 645, 45.6741°N, 66.8661°W, 26.IV-10.V.2010, R. Webster & C. MacKay, old mixed forest with red and white spruce, red and white pine, balsam fir, eastern white cedar, red maple, and *Populus* sp., Lindgren funnel traps (2, AFC).

###### Collection and habitat data.

*Dendrophagus cygnaei* was found in various forest types in New Brunswick, including hardwood forests, an old red oak (*Quercus rubra* L.) forest, mixed forests, a red spruce (*Picea rubens* Sarg.) forest, an old (180-year-old) red pine (*Pinus resinosa* Ait.) forest, an old-growth northern hardwood forest, an old-growth white spruce (*Picea glauca* (Moench) Voss) and balsam fir (*Abies balsamea* (L.) Mill.) forest, and an old-growth eastern white cedar (*Thuja occidentalis* L.) forest. Adults were collected from under bark of *Populus* and sugar maple (*Acer saccharum* Marsh.). This species was commonly collected in Lindgren funnel traps at most sites where these traps were deployed. Adults were collected during April, May, June, and July.

###### Distribution in Canada and Alaska.

AK, BC, AB, MB, ON, QC, NB, NS (Bousquet 1991; Majka 2008). The New Brunswick record of *Dendrophagus cygnaei* reported by Majka (2008) was based on a specimen collected by W. McIntosh from Saint John in June of a year predating 1910. The above records provide the first recent records of this species from the province.

**Map 1.**
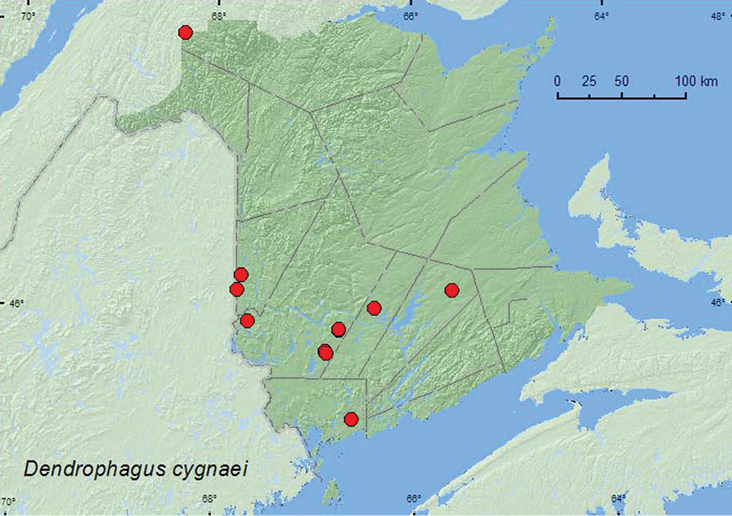
Collection localities in New Brunswick, Canada of *Dendrophagus cygnaei*.

#### Subfamily Silvaninae Kirby, 1837

##### Silvanus muticus

Sharp**

http://species-id.net/wiki/Silvanus_muticus

Map 2

###### Material examined.

**New Brunswick, Carleton Co.**, Jackson Falls, Bell Forest, 46.2200°N, 67.7231°W, 12.VII.2006, 25.VII.2007, R. P. Webster, mature hardwood forest, u.v. light (7, NBM, RWC). **Sunbury Co.**, Acadia Research Forest, 45.9866°N, 66.3841°W, 2–8.VI.2009, R. Webster & M.-A. Giguère, mature (110-year-old) red spruce forest with scattered red maple and balsam fir, Lindgren funnel trap (1, AFC). **York Co.**, Fredericton, at Saint John River, 45.9588°N, 66.6254°W, 7.VI.2005, R. P. Webster, river margin in flood debris (1, RWC); Charters Settlement, 45.8340°N, 66.7450°W, 16.VIII.2006, R. P. Webster, mixed forest, beating (dead) birch branches with dead dried leaves (4, RWC).

###### Collection and habitat data.

*Silvanus muticus* was collected in a mature hardwood forest, a mature (110-year-old) red spruce forest, and a mixed forest. Adults were collected by beating dead white birch (*Betula papyrifera* Marsh.) branches that had dead, dried leaves, sifting flood debris on a river margin, and at an ultraviolet light. Adults were collected during June, July, and August.

###### Distribution in Canada and Alaska.

BC, ON, QC, **NB** (Bousquet 1991).

**Map 2.**
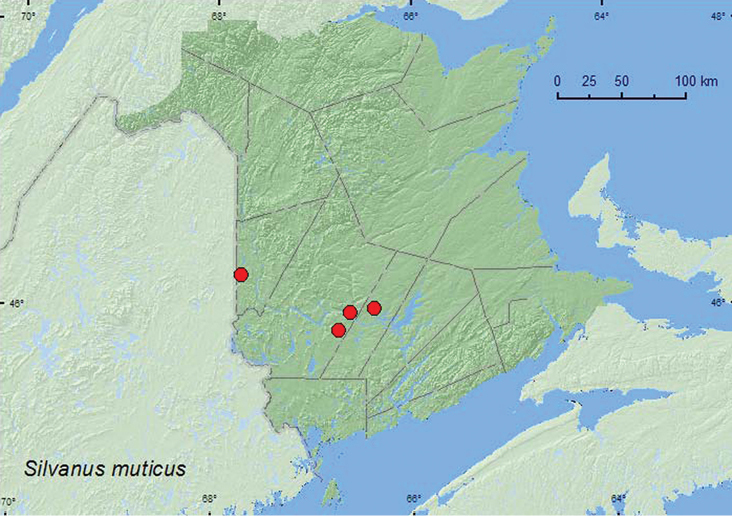
Collection localities in New Brunswick, Canada of *Silvanus muticus*.

##### Ahasverus longulus

(Blatchley, 1910)**

http://species-id.net/wiki/Ahasverus_longulus

Map 3

###### Material examined.

**New Brunswick, York Co.**, Charters Settlement, 45.8428°N, 66.7279°W, 23.VI.2004, R. P. Webster & H. Goulet, small sedge marsh in moist grass litter (1, RWC); Charters Settlement, 45.8267°N, 66.7343°W, 14.V.2005, R. P. Webster, margin of *Carex* marsh/fen, in sphagnum and leaf litter at base of tree (1, RWC); 9.0 km W of Tracy off Rt. 645, 45.6889°N, 66.8002°W, 5.IV.2010, R. P. Webster, old beaver flowage, in grass litter on clay soil near small stream (1, RWC).

###### Collection and habitat data.

*Ahasverus longulus* was found in *Carex* marshes and in an old beaver (*Castor canadensis* Kuhl) flowage. Adults were sifted from grass litter and sphagnum and leaf litter at the base of a tree during May and June.

###### Distribution in Canada and Alaska.

ON, QC, **NB** (Bousquet 1991). *Ahasverus longulus* was included in Bousquet’s (1991) checklist. However, no voucher specimens or published records could be found to support the record, and consequently, Makja (2008) removed the species from the faunal list of New Brunswick. In view of the above records, the species is re-instated to the faunal list of the province.

**Map 3.**
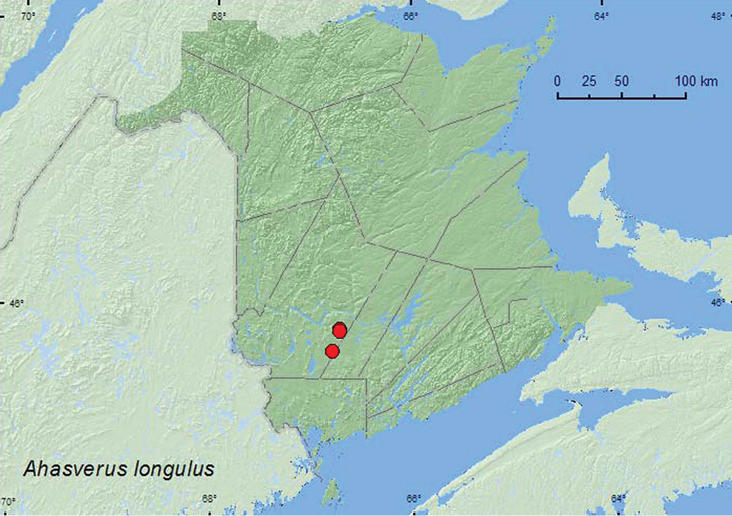
Collection localities in New Brunswick, Canada of *Ahasverus longulus*.

#### Family Laemophloeidae Ganglbauer, 1899

Thomas (2002b) presented a general overview of the family Laemophloeidae (lined flat bark beetles) of North America. Most species live under bark, as their common name implies and probably feed on fungi, although species in a few genera may be predacious on Scolytinae (Thomas 2002b). Several species are important stored-product pests. Bousquet (1991) reported only *Laemophloeus biguttatus* (Say) from New Brunswick, and Majka (2008) later added *Cryptolestes pusillus* (Schönherr) and *Laemophloeus fasciatus* Melsheimer to the faunal list. Here, we add five additional species of this family, bringing the total number of species of this family known from New Brunswick to eight (Table 1).

##### Charaphloeus convexulus

(LeConte, 1879)

http://species-id.net/wiki/Charaphloeus_convexulus

Map 4

###### Material examined.

**New Brunswick, Carleton Co.**, Jackson Falls, Bell Forest, 46.2200°N, 67.7231°W, 23–28.IV.2009, 28.IV–9.V.2009, 9–14.V.2009, R. Webster & M.-A. Giguère, mature hardwood forest, Lindgren funnel traps (8, AFC, RWC). **Queens Co.**, Cranberry Lake P.N.A, 46.1125°N, 65.6075°W, 24.IV-5.V.2009, 5–12.V.2009, 21–27.V.2009, R. Webster & M.-A. Giguère, old red oak forest, Lindgren funnel traps (10, AFC, NBM, RWC). **Sunbury Co.**, Acadia Research Forest, 45.9866°N, 66.3841°W, 25.V–2.VI.2009, R. Webster & M.-A. Giguère, mature (110 year-old) red spruce forest with scattered red maple and balsam fir, Lindgren funnel trap (1, RWC). **York Co.**, Charters Settlement, 45.8395°N, 66.7391°W, 6.V.2008, R. P. Webster, mixed forest, in flight during warm (20°C) evening (1, RWC); same locality data and collector but 23–27.V.2009, mixed forest, Lindgren funnel trap (1, RWC); 15 km W of Tracy off Rt. 645, 45.6848°N, 66.8821°W, 25.IV–4.V.2009, R. Webster & M.-A. Giguère, old red pine forest, Lindgren funnel trap (1, AFC);14 km WSW of Tracy, S of Rt. 645, 45.6741°N, 66.8661°W, 25.V–2.VI.2010, R. Webster & C. MacKay, old mixed forest with red and white spruce, red and white pine, balsam fir, eastern white cedar, red maple, and *Populus* sp., Lindgren funnel trap (1, AFC).

###### Collection and habitat data.

*Charaphloeus convexulus* was found in various forest types in New Brunswick, including mature hardwood forests, an old red oak forest, mixed forests, a red spruce forest, and an old-growth red pine forest. However, this species was most frequently collected in hardwood forests. Most adults were captured in Lindgren funnel traps. One individual was captured with an aerial net during a warm evening. Adults were collected during April, May, and June (most during May). This species usually occurs under bark (Thomas 1993).

###### Distribution in Canada and Alaska.

ON, **NB**, NS (Bousquet 1991; Majka 2008).

**Map 4.**
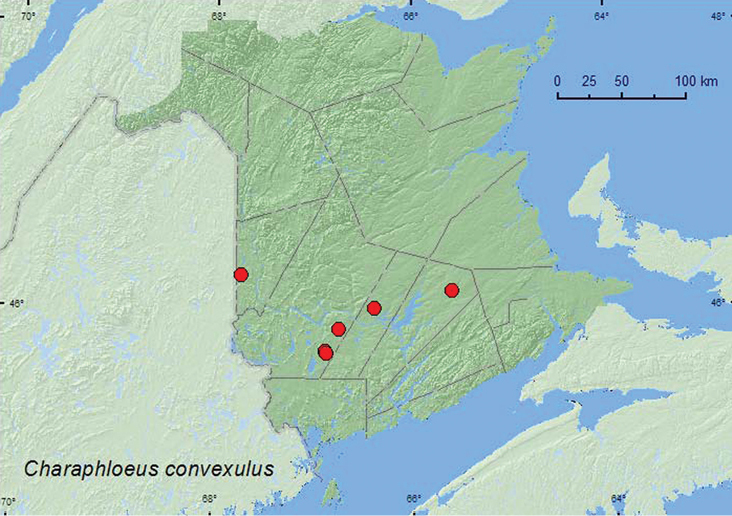
Collection localities in New Brunswick, Canada of *Charaphloeus convexulus*.

##### Charaphloeus undescribed species (near adustus)

Map 5

###### Material examined.

**New Brunswick, Carleton Co.**, Meduxnekeag Valley Nature Preserve, 46.1890°N, 67.6766°W, 8.VI.2008, R. P. Webster & M.-A. Giguère, floodplain forest, on flowers of *Prunus virginiana* (2, RWC). **Charlotte Co.**, 10 km NW of New River Beach, 45.2110°N, 66.6170°W, 17–31.V.2010, R. Webster & C. MacKay, old growth eastern white cedar forest, Lindgren funnel trap (1, RWC). **Queens Co.**, Grand Lake Meadows P.N.A., 45.8227°N, 66.1209°W, 10–31.V.2010, 31.V-15.VI..2010, R. Webster & C. MacKay, old silver maple forest with green ash and seasonally flooded marsh, Lindgren funnel traps (2, RWC); Cranberry Lake P.N.A, 46.1125°N, 65.6075°W, 13–25.V.2011, M. Roy & V. Webster, old red oak forest, Lindgren funnel trap (1, RWC). **Sunbury Co.**, Acadia Research Forest, 45.9866°N, 66.3841°W, 24–30.VI.2009, R. Webster & M.-A. Giguère, mature (110 year-old) red spruce forest with scattered red maple and balsam fir, Lindgren funnel trap (1, RWC). **York Co.**, 15 km W of Tracy off Rt. 645, 45.6848°N, 66.8821°W, 4–11.V.2009, R. Webster & M.-A. Giguère, old red pine forest, Lindgren funnel trap (1, RWC); Charters Settlement, 45.8395°N, 66.7391°W, 2.V.2010, R. P. Webster, mixed forest, in flight during warm (20°C) evening, 16:30–20:00 h (1, RWC).

###### Collection and habitat data.

Most adults were captured in Lindgren funnel traps in a silver maple (*Acer saccharinum* L.) forest, an old-growth eastern white cedar forest, a red spruce forest, an old red pine forest, and a mixed forest. A few adults were collected from flowers of *Prunus virginiana* L. in a floodplain forest, and one was collected with an aerial net during an evening flight. Adults were collected during May and June.

###### Distribution in Canada and Alaska.

**NB**, NS (Majka 2008). This undescribed species [see key in Downie and Arnett (1996: 1001) on how to separate from related species] was newly recorded from Canada by Majka (2008) from a specimen collected in Debert, Colchester Co., Nova Scotia.

**Map 5.**
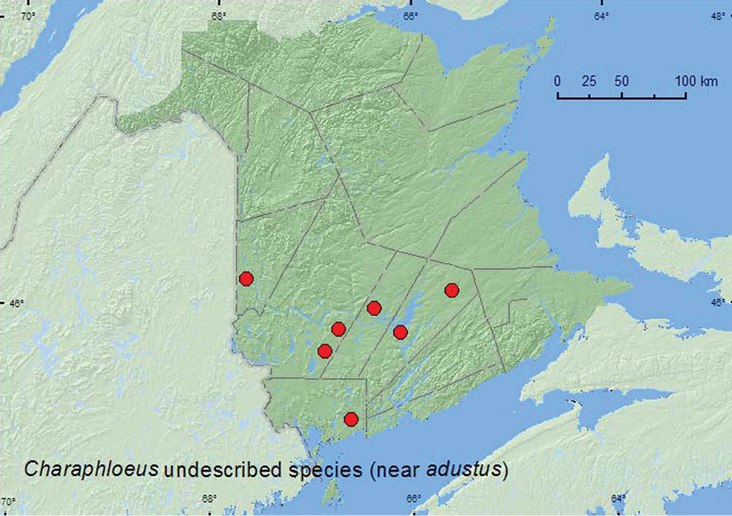
Collection localities in New Brunswick, Canada of *Charaphloeus* undescribed species (near *adustus*).

##### Placonotus zimmermanni

(LeConte, 1854)

http://species-id.net/wiki/Placonotus_zimmermanni

Map 6

###### Material examined.

**New Brunswick, Carleton Co.**, Jackson Falls, Bell Forest, 46.2200°N, 67.7231°W, 14–20.V.2009, R. Webster & M.-A. Giguère, mature hardwood forest, Lindgren funnel trap (1, RWC). **Queens Co.**, Grand Lake Meadows P.N.A., 45.8227°N, 66.1209°W, 27.VI–5.VII.2011, M. Roy & V. Webster, old silver maple forest and seasonally flooded marsh, Lindgren funnel traps (2, RWC).

###### Collection and habitat data.

The New Brunswick specimens of *Placonotus zimmermanni* were captured in Lindgren funnel traps deployed in a mature hardwood forest with American beech (*Fagus grandifolia* Ehrh.) and sugar maple and in an old silver maple swamp. Adults at the latter site were captured in traps in the forest canopy. Majka (2008) reported this species from a red oak forest (window trap) in Nova Scotia. Thomas (1993) reported collecting this species from under bark of dead hardwoods, including oaks, in association with ascomycete fungi.

###### Distribution in Canada and Alaska.

MB, ON, QC, **NB**, NS (Bousquet 1991; Majka 2008).

**Map 6.**
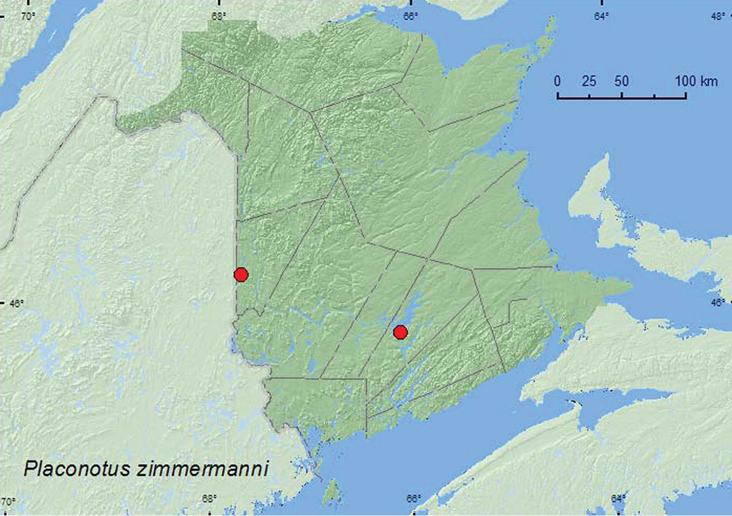
Collection localities in New Brunswick, Canada of *Placonotus zimmermanni*.

##### Leptophloeus angustulus

(LeConte, 1866)

http://species-id.net/wiki/Leptophloeus_angustulus

Map 7

###### Material examined.

**New Brunswick, Carleton Co.**, Jackson Falls, Bell Forest, 46.2200°N, 67.7231°W, 19–28.VII.2008, R. P. Webster, mature hardwood forest, Lindgren funnel trap (1, AFC). **Queens Co.**, Grand Lake Meadows P.N.A., 45.8227°N, 66.1209°W, 12–26.VII.2010, R. Webster & C. MacKay, old silver maple forest with green ash and seasonally flooded marsh, Lindgren funnel trap (1, RWC); Cranberry Lake P.N.A, 46.1125°N, 65.6075°W, 7–13.VII.2011, 13–20.VII.2011, 20.VII-4.VIII.2011, 4–18.VIII.2011, M. Roy & V. Webster, old red oak forest, Lindgren funnel traps (5, NBM, RWC).

###### Collection and habitat data.

This species was captured in Lindgren funnel traps deployed in a silver maple forest, a sugar maple and American beech forest, and an old red oak forest. Two of the specimens were captured in traps in the forest canopy. Adults in New Brunswick were captured during July. This species is apparently a predator of Scolytinae and has been collected from oaks infested with *Pseudopityopththorus pruinosus* (Eichoff) in Oklahoma (Thomas 1993) and from a window trap deployed in a red oak infested with *Pseudopityopththorus minutissimus* (Zimmerman) in Nova Scotia (Majka and Chandler 2009).

###### Distribution in Canada and Alaska.

NS, **NB** (Majka and Chandler 2009). Majka and Chandler (2009) reported this species for the first time for Nova Scotia and Canada (Bridgewater) and the New England States (Odiorne Point, New Hampshire).

**Map 7.**
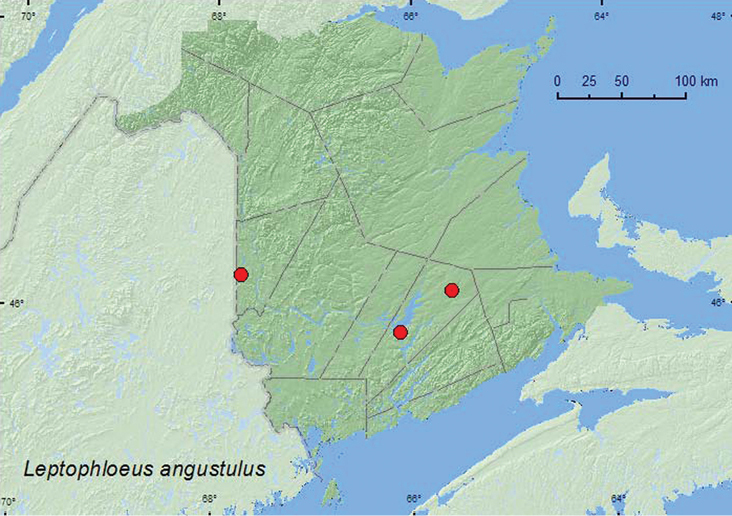
Collection localities in New Brunswick, Canada of *Leptophloeus angustulus*.

##### Leptophloeus sp.**

Map 8

###### Material examined.

**New Brunswick, Restigouche Co.**, Dionne Brook P.N.A., 47.9064°N, 68.3441°W, 31.V-15.VI.2011, M. Roy & V. Webster, old-growth white spruce and balsam fir forest, Lindgren funnel traps (2, RWC).

###### Collection and habitat data.

Both adults were captured during June in Lindgren funnel traps deployed in an old-growth white spruce and balsam fir forest.

###### Distribution in Canada and Alaska.

BC, AB, YT, QC, **NB** (Bousquet 1991). There are specimens in the CNC from AB and YT (Bousquet, personal communication). Bousquet (1991) reported this species as *Leptophloeus alternans* (Erichson) in the *Checklist of the Beetles of Canada*. However, Thomas (1993) considered this to be an undescribed species. See Thomas (1993) for additional comments on the status of this species. There are at least two additional undescribed species from the western United States (Thomas 1993).

**Map 8.**
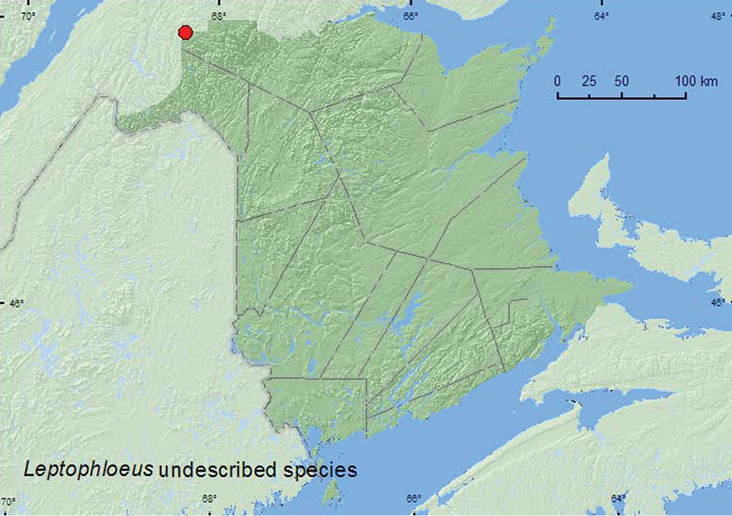
Collection localities in New Brunswick, Canada of *Leptophloeus* undescribed species.

## Supplementary Material

XML Treatment for Dendrophagus cygnaei

XML Treatment for Silvanus muticus

XML Treatment for Ahasverus longulus

XML Treatment for Charaphloeus convexulus

XML Treatment for Charaphloeus

XML Treatment for Placonotus zimmermanni

XML Treatment for Leptophloeus angustulus

XML Treatment for Leptophloeus
